# Remnant kelp bed refugia and future phase-shifts under ocean acidification

**DOI:** 10.1371/journal.pone.0239136

**Published:** 2020-10-09

**Authors:** Scott D. Ling, Christopher E. Cornwall, Bronte Tilbrook, Catriona L. Hurd

**Affiliations:** 1 Institute for Marine & Antarctic Studies, University of Tasmania, Hobart, Tasmania, Australia; 2 School of Biological Sciences, Victoria University of Wellington, Kelburn, Wellington, New Zealand; 3 CSIRO Oceans & Atmosphere, Hobart, Tasmania, Australia; 4 Antarctic Climate and Ecosystems Co-operative Research Centre, University of Tasmania, Hobart, Tasmania, Australia; University of Sydney, AUSTRALIA

## Abstract

Ocean warming, ocean acidification and overfishing are major threats to the structure and function of marine ecosystems. Driven by increasing anthropogenic emissions of CO_2_, ocean warming is leading to global redistribution of marine biota and altered ecosystem dynamics, while ocean acidification threatens the ability of calcifying marine organisms to form skeletons due to decline in saturation state of carbonate Ω and pH. In Tasmania, the interaction between overfishing of sea urchin predators and rapid ocean warming has caused a phase-shift from productive kelp beds to overgrazed sea urchin barren grounds, however potential impacts of ocean acidification on this system have not been considered despite this threat for marine ecosystems globally. Here we use automated loggers and point measures of pH, spanning kelp beds and barren grounds, to reveal that kelp beds have the capacity to locally ameliorate effects of ocean acidification, via photosynthetic drawdown of CO_2_, compared to unvegetated barren grounds. Based on meta-analysis of anticipated declines in physiological performance of grazing urchins to decreasing pH and assumptions of nil adaptation, future projection of OA across kelp-barrens transition zones reveals that kelp beds could act as important pH refugia, with urchins potentially becoming increasingly challenged at distances >40 m from kelp beds. Using spatially explicit simulation of physicochemical feedbacks between grazing urchins and their kelp prey, we show a stable mosaicked expression of kelp patches to emerge on barren grounds. Depending on the adaptative capacity of sea urchins, future declines in pH appear poised to further alter phase-shift dynamics for reef communities; thus, assessing change in spatial-patterning of reef-scapes may indicate cascading ecological impacts of ocean acidification.

## Introduction

Ocean warming (OW) and acidification (OA) pose major threats to the world’s marine ecosystems, with both occurring because of increasing anthropogenic emissions of CO_2_ [1]. OW has already impacted marine ecosystems via wholesale redistribution of biodiversity [2] and is particularly dire when it impacts foundational biogenic reef habitats, for example, mass coral bleaching [3] and die-backs of kelp beds [4–7]. While OW impacts are realized globally [2], OA is less well understood. However, the co-occurrence of both these climate change stressors, along with interactions with non-climate stressors such as pervasive impacts of overfishing [8, 9], will cause further alteration to marine ecosystems in the near future. Given observed impacts of climate change on marine ecosystems and associated human-wellbeing [2], there is an urgent need to identify hotspots of further risk [10, 11] and define the mechanisms of resilience [8] and/or refugia [12–15].

Kelp beds could provide local refuge from OA and safeguard the species and community occurring within [13, 16, 17]. Kelps modify pH via photosynthetic drawdown of dissolved inorganic carbon [18–20], and also provide food for herbivorous species, both of which may locally ameliorate the effects of OA on inhabitant species [12, 13, 21]. Indeed, a key-driver of kelp bed loss globally is herbivory, which is chiefly determined by the abundance of sea urchins capable of overgrazing and affecting a phase-shift from kelp beds to urchin barren grounds [22, 23]. As calcifying organisms, sea urchins are thus a pivotal reef organism vulnerable to OA and an extensive literature now documents negative effects of altered pH on urchins, particularly within laboratory settings where 95% of the 141 empirical OA-urchin studies have been conducted (S1 Fig in S1 File). However, the impact of OA on sea urchins and broader ecological feedbacks have rarely been considered in natural ecosystems/in the field. Only 5% of articles assessing the impacts of OA are field-based, exclusive of studies conducted at atypical volcanic CO_2_ vent systems (S1 Fig in S1 File). This indicates a greater need to integrate the understanding gained in the laboratory within the context of processes and patterns occurring in the field. Identifying possible ecological feedbacks between sea urchins and their kelp prey under OA is therefore important for anticipating and detecting change in the nature of phase-shifts for reef ecosystems into the future [24].

**Fig 1:**
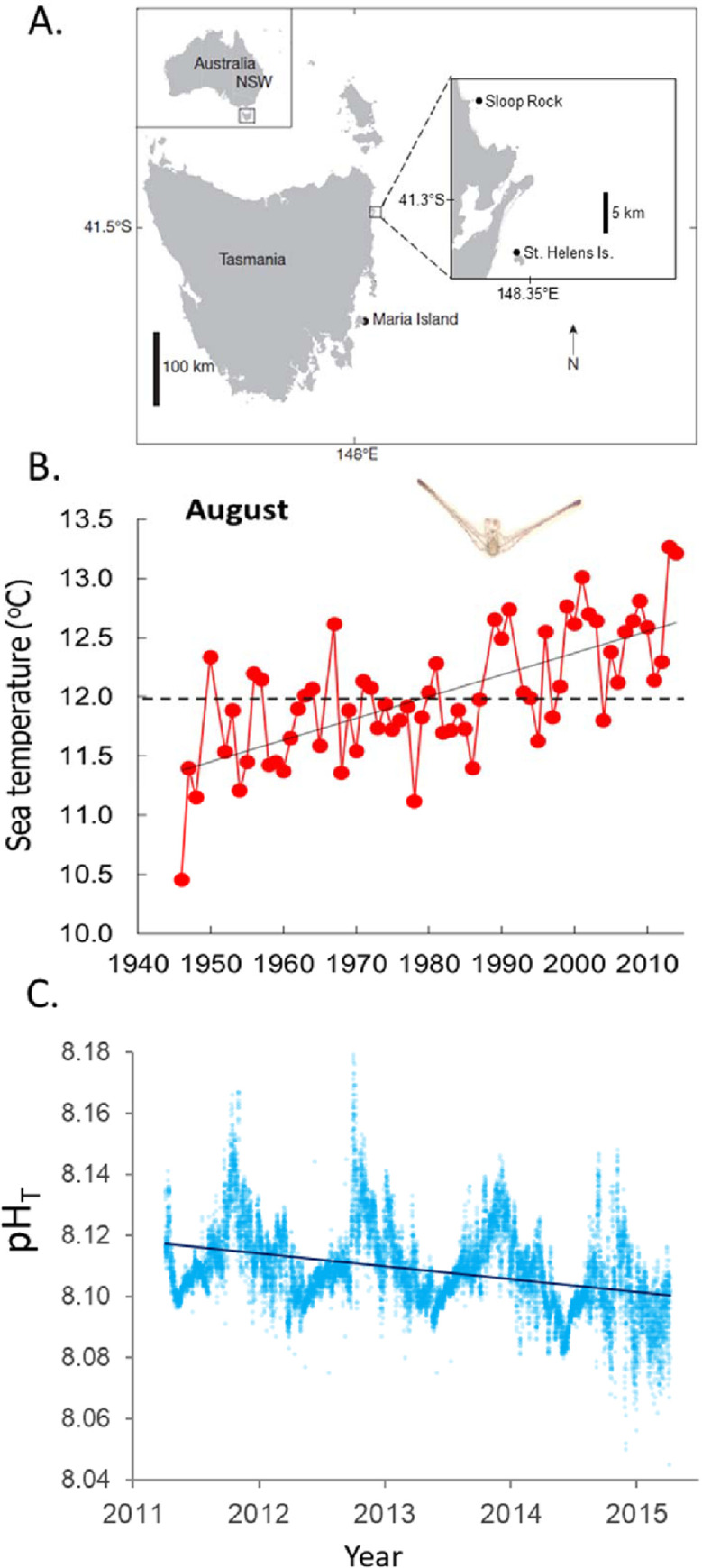
Marine climate change trends in eastern Tasmania. (A) Location of study sites at St. Helens in Tasmania, south-eastern Australia; long-term oceanographic station offshore from Maria Island also shown. (B) 70 year trend in August sea temperature in eastern Tasmania as sampled at the Maria Is. oceanographic station (1944–2014), horizontal line is lower temperature threshold for successful larval development of *Centrostephanus rodgersii* (inset) as spawned during August [28]. (C) Trend in ocean pH as sampled every 2 hours from April 2011 –April 2015 at the Maria Island National Reference Station, which is part of long-term pH decline for this region. Data for (B) and (C) courtesy of the Integrated Marine Observing System/ CSIRO.

On the east coast of Tasmania, Australia, interaction between climate-warming and overfishing of large urchinivorous rock lobsters (*Jasus edwardsii*) from kelp beds has reduced resilience of kelp beds against overgrazing by the sea urchin (*Centrostephanus rodgersii*) [8, 22]. The tropical-family diadematid *C*. *rodgersii* has extended its range poleward from New South Wales to the historically cool Tasmanian waters in response to rapid ocean warming (Fig 1A), which has occurred at approximately four times the global average of ocean warming [25, 26]. Given ongoing ocean warming and increasingly suitable conditions for larval development of *C*. *rodgersii* in Tasmanian waters (Fig 1B), and the low resilience of resident *Ecklonia radiata* kelp beds due to the absence of large functionally important sea urchin predators extirpated by intense fishing [8], the urchin population is expanding to create new “incipient” barrens patches within kelp beds while existing barrens patches are coalescing into extensive barren grounds which impact reef-based fisheries and biodiversity across large scales [22, 27]. With the increasing threat of OA, which is confirmed by a regional decrease in ocean pH (Fig 1C), it is important to understand how the combined stressors of ocean warming, overfishing and OA will conspire to alter reef ecosystem dynamics.

Here we explore well known overgrazing phase-shift dynamics for the eastern Tasmanian reef systems in the context of projected pH decline under OA to 2100 under the business-as-usual scenario (RCP8.5 [1]). We hypothesized that pH would be higher within kelp beds than on extensive urchin barren grounds due to photosynthetic drawdown of dissolved inorganic carbon by kelps. We test this hypothesis by comparing measurements of seawater pH within kelp beds and on urchin barrens using deployment of automated loggers in combination with point measurements using bottle sampling across kelp barrens interfaces. Furthermore, to anticipate possible future dynamics of kelp/ urchin barrens phase-shifts, we project observed differences in the pH gradient across the kelp bed/ barrens interface under OA to 2100 to gauge possible declines in sea urchin performance based on meta-analysis of laboratory studies. We then derive simple spatial rules of physicochemical feedback between grazing urchins and kelp prey under different scenarios of urchin adaptation to low pH and use spatially explicit cellular automata to explore possible future reef-scape patterns emerging under OA.

## Results

### Kelp bed elevation of local pH relative to barren grounds

**Fig 2:**
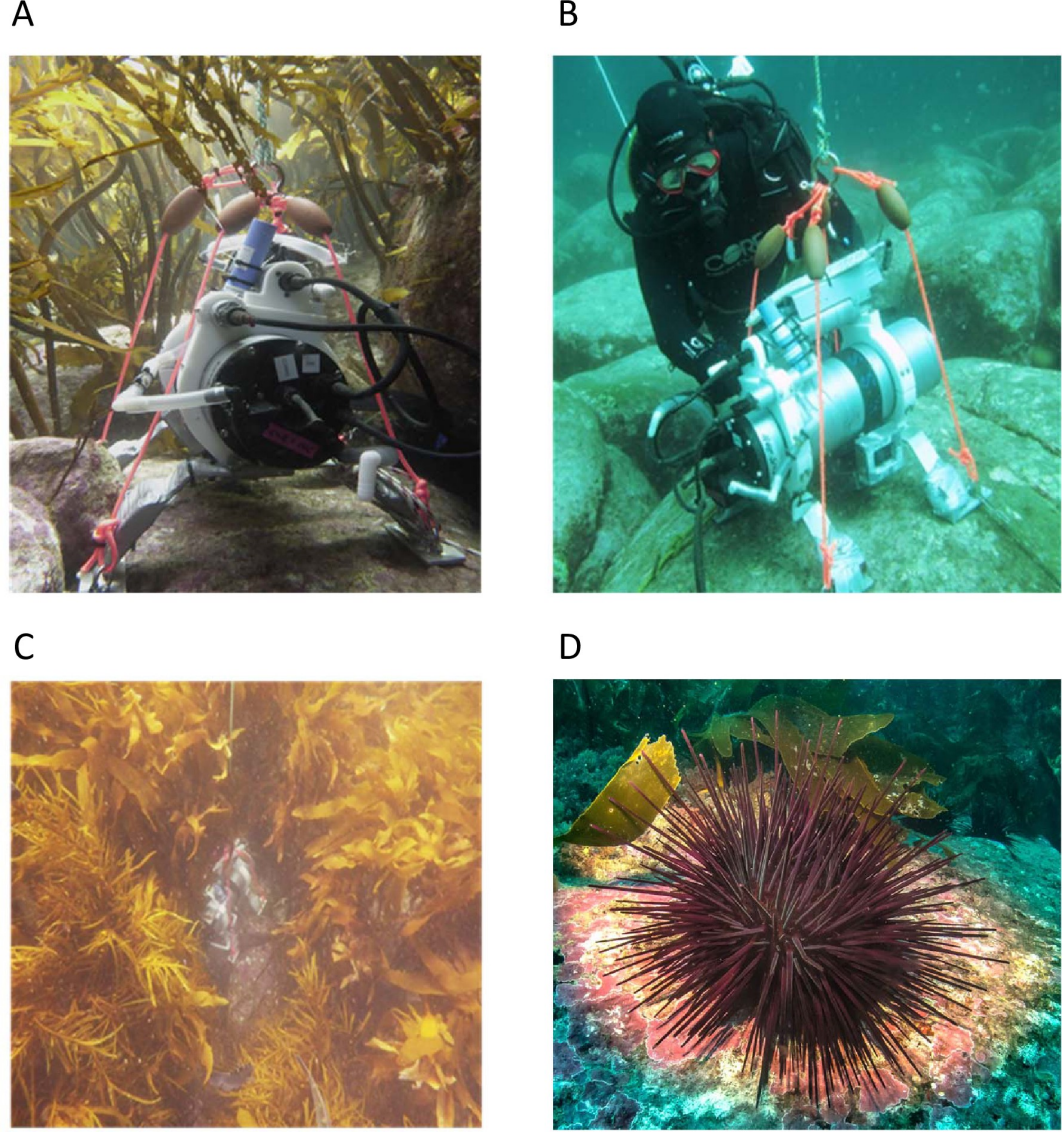
Sampling of pH in kelp beds and on sea urchin barrens ground at St. Helens, northeast Tasmania. Automated SeaPhOx sampling within kelp beds (A and C) and on barrens ground (B); panel (D) shows the sea urchin, *Centrostephanus rodgersii*, grazing at the edge of a kelp bed (Photographic credit: A, C, D Scott Ling; B Christopher Cornwall).

**Fig 3:**
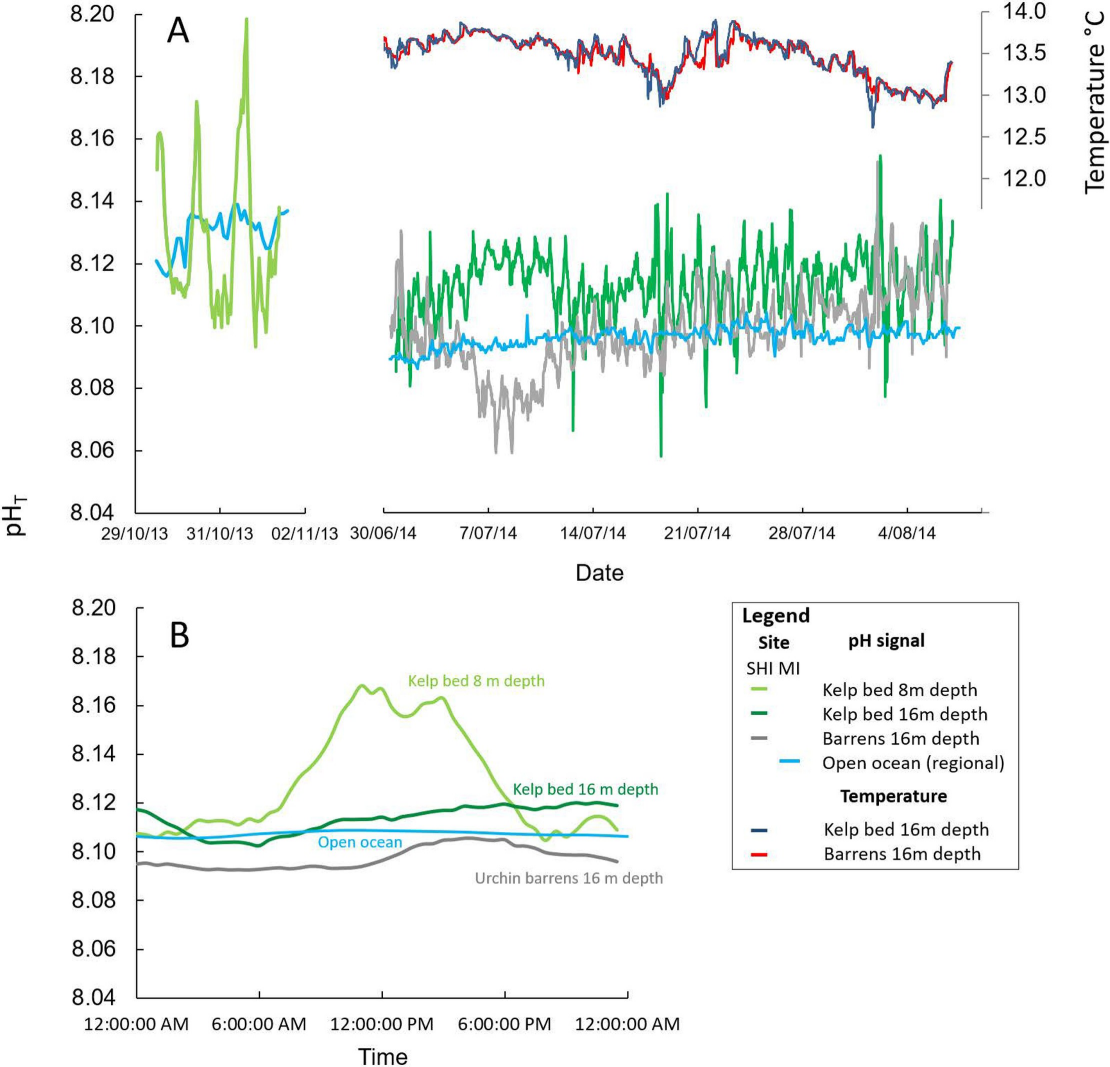
Kelp bed elevation of local pH relative to barrens ground. (A) pH traces from automated loggers in kelp beds (green) and urchin barrens (grey) at St. Helens Island (SHI); for shallow kelp beds (LHS plot, from 29^th^ to 31^st^ Oct 2013; see legend), and for deeper (16 m depth) kelp beds and barrens ground (RHS plot, from 5^th^ June to 7^th^ Aug 2014); temperature traces in kelp beds and barrens (navy blue and red, respectively) and open ocean pH (blue) are also shown, see legend. (B) Composite plot of mean diurnal pH for shallow kelp beds (Oct 2013), deep kelp beds (June-Aug 2014), regional open ocean surface pH (June-Aug 2014 at Maria Island (MI)), and urchin barrens (June-Aug 2014); see S3 Fig for seaonality in regional open ocean surface pH and temperature in S1 File.

pH measurements were obtained within kelp beds and on urchin barren grounds (see full habitat descriptions in S1 Table and S2 Fig in S1 File) using automated SeapHOx sensors deployed over 82 days (Fig 2A–2D), in combination with Niskin bottle samples used to map pH across the transitional zone between the alternative reef states. Consistent differences in pH were observed between the reef states with a distinct elevation of pH occurring within the kelp beds relative to that observed on barren grounds (Fig 3A). This occurred despite both reef states experiencing the same bulk water mass as revealed by temperature traces (Fig 3A). Observed pH variability in kelp beds and on barren grounds was consistently higher than the open ocean signal obtained over the same period from a long-term mooring station in the region (Fig 3A). Furthermore, the mean daily trend in open ocean pH showed very little variability compared to sharp diel variability evident for reefs, which peaked at midday for shallow kelp beds and in late afternoon for deeper kelp kelps and barren grounds (Fig 3B). Importantly, the mean daily trend in pH on barren grounds was consistently observed to occur below that observed for the open ocean (Fig 3B), with minimum pH values occurring on sea urchin barrens at night during winter when pH is both seasonally (S3 Fig in S1 File) and diurnally low (Fig 3B). In contrast, peak pH was observed during the middle of the day within dense shallow kelp beds (Fig 3B), which is consistent with highest levels of kelp biomass and the period of maximal daily photosynthetic activity (S2 Fig in S1 File).

### Spatial patterns in pH across kelp bed/ barren ground interfaces

**Fig 4:**
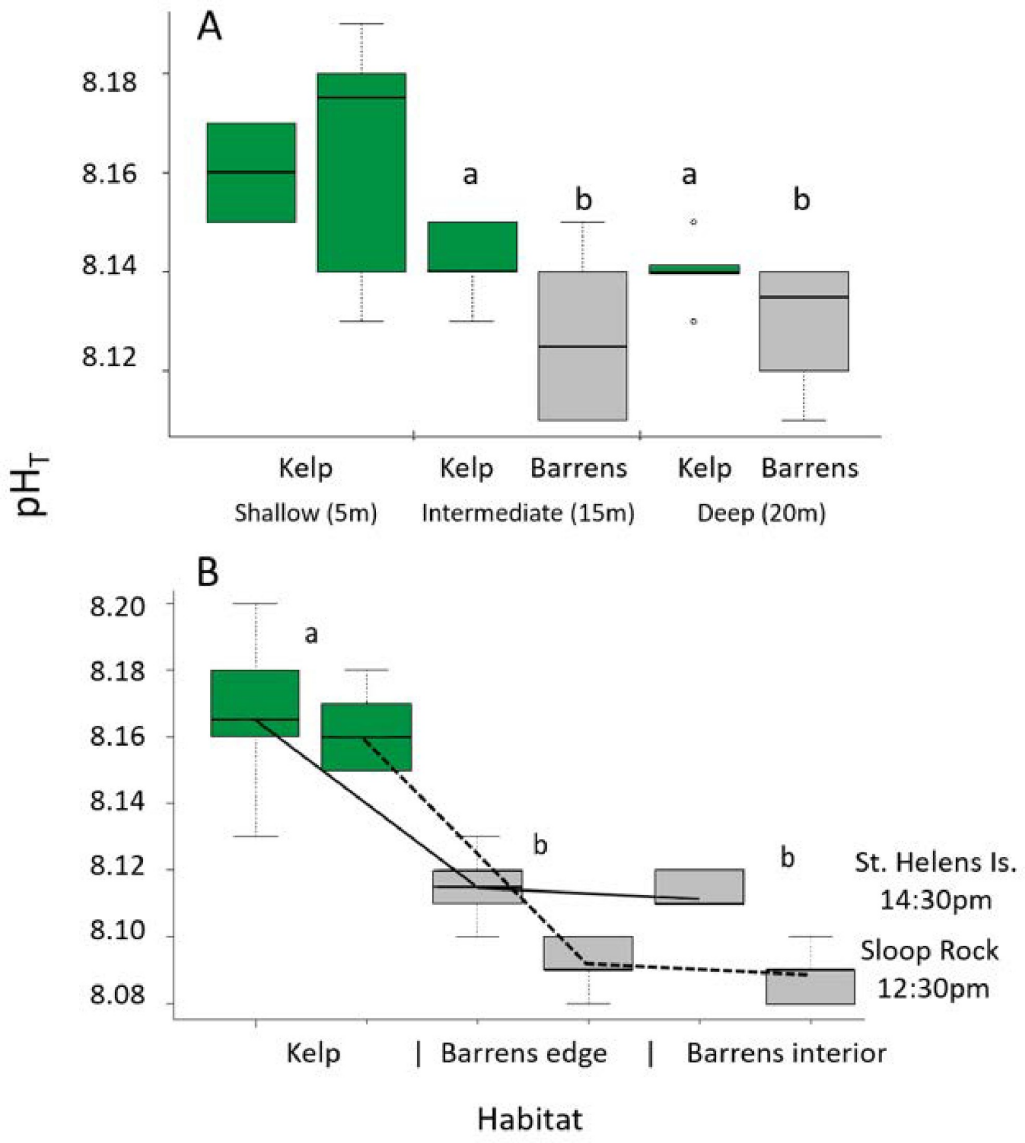
Spatial patterns in pH across kelp bed/ barren ground interfaces. (A) pH within shallow kelp beds and on sea urchin barrens in intermediate and deep reefs at St. Helens Island during Aug 2014, data are means of n = 6 replicate bottle samples (note that barrens are not currently found on shallow reefs at this site and at this depth generally in eastern Tasmania); (B) pH within kelp beds, barrens edge and barrens interior at St. Helens Island and Sloop Rock (St. Helens, north eastern Tasmania) during Oct 2013, data are means of n = 6 replicate bottle samples. Different letters indicate significantly different pH between habitats (see S2 Table for analyses of variance in S1 File).

The reduced pH on barrens ground occurred independently of depth, as revealed by bottle-sampling across multiple reefs (Fig 4A). While dense kelp beds in the shallows can create thicker benthic boundary layers and contain a relatively small volume of seawater over which local water chemistry is altered [29, 30], kelp beds in deeper water also elevated pH above that on barrens ground at the same depth; albeit at a reduced rate consistent with reduced kelp biomass and light levels (for kelp biomass see S2 Fig in S1 File). The profile in pH across the kelp bed/ barrens interface revealed a progressive decline across the transitional zone with minimal readings occurring in the interior of the barren grounds (Fig 4B).

### Projecting pH gradients and phase-shift under ocean acidification

**Fig 5:**
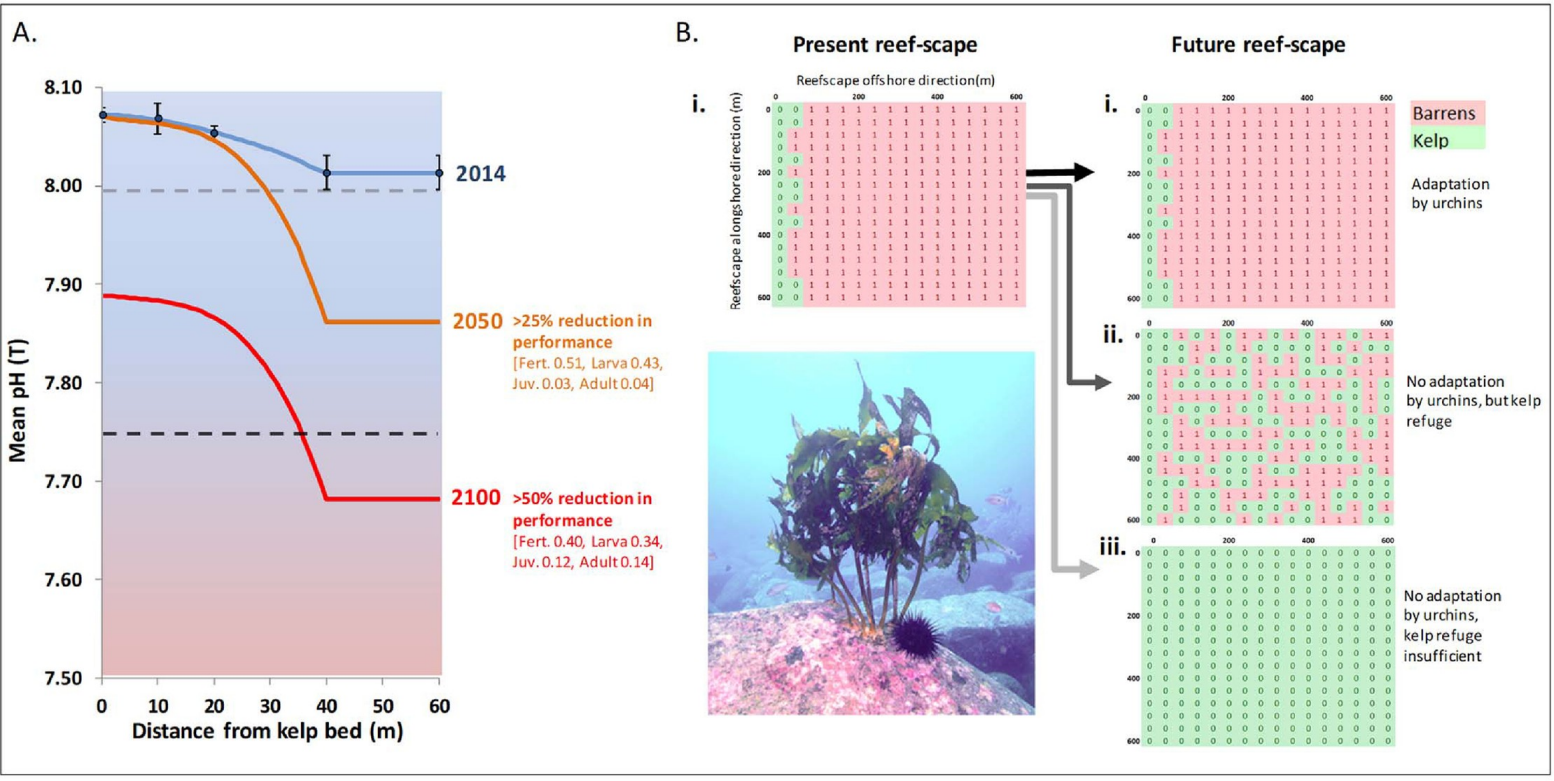
Modelling future phase-shifts under different scenarios of adaptation by sea urchins to ocean acidification. (A) Spatial dynamics of pH proximate to kelp beds in 2014 (means ± SE, n = 4 per sample position) and projected under OA business as usual (RCP8.5) for years 2050 and 2100; dashed horizontal lines indicate thresholds for compromised performance of sea urchins at 25% (light grey) and 50% (black) (S5 Fig in S1 File); numbers in parentheses indicate proportional contribution by each life-history stage to the compounded decline in sea urchin performance across life-history stages. (B) Conceptualization of present-day (i) and future reef-scapes under OA (green cells “0” = kelp; pink cells “1” = urchin barren); (i) maintenance of present-day configuration due to adaptation by urchins, i.e. extensive barrens and narrow fringing kelp beds in the shallows; (ii) emergent mosaicked kelp/ barrens future simulated by cellular automaton of barrens persistence within 40 m of kelp refuge with nil adaptation by urchins (from 4A.); (iii) extensive kelp beds due to nil adaptation by urchins and insufficient kelp refuge from OA. Simulated reef-scapes run alongshore in the direction of the y-axis; and offshore in the direction of the x-axis; and are a total of 640 m by 640 m with each cell 40 m by 40 m. Inset image shows urchin at the base of a remnant kelp patch at St. Helens Island study site.

Given the present-day gradient in pH across the kelp bed/ barrens interface, projections of OA across this interface (under business-as-usual CO_2_ emissions projection—RCP8.5) indicates the potential for an increasing ameliorating effect of kelp beds on pH through time (Fig 5A). Despite predictions of ongoing decline in the pH of eastern Tasmanian coastal waters, concurrent warming of mean annual temperature from ~14°C by ~+4°C by 2100 [31, 32] was not considered to negatively affect either the kelp or the sea urchin species directly as both species are found in northern New South Wales in coastal waters with an average annual temperature of ~22°C. Thus, temperature was not explicitly considered. Under the assumption of nil adaptation to OA, projections indicate that maintenance of barrens beyond ~40 m from kelp refugia could become increasingly difficult due to reducing physiological performance of sea urchins (Fig 5A). Spatially explicit simulation of the ameliorating effect of kelp, using cellular automata seeded with the present-day reef-scape (Fig 5Bi), revealed the emergence of a novel and stable mosaicked reef-scape configuration under OA (Fig 5Bii). Alternatively, if urchins sufficiently adapt to low pH conditions by 2100 and/ or impacts are less severe than anticipated from laboratory experiments, then present-day barren grounds will persist (Fig 5Bi–i). However, if the effects of OA are severe and grazing urchins do not adapt, urchins may become locally diminished and widespread kelp bed recovery could occur (Fig 5Biii).

## Discussion

Increasing atmospheric CO_2_ driven by anthropogenic climate change is leading to ocean warming and ocean acidification, which along with non-climate anthropogenic stressors is increasingly threatening the structure and functioning of marine ecosystems. Occurring within a globally significant hotspot of ocean warming, interactive effects between overfishing and rapid warming has enabled a range-extending sea urchin to overgraze Tasmanian kelp beds resulting in impoverished barren grounds [8]. Within this altered reef-scape, we show that under projected conditions of OA that remnant kelp can ameliorate low pH via photosynthetic drawdown of CO_2_, thus providing calcifying organisms, including urchins themselves, with a potential refugium. In the absence of adaptation by urchins, simulation using spatial automata indicate novel phase-shift dynamics whereby spatially complex feedbacks between kelp, sea urchins and the chemical environment lead to a novel and stable mosaicked reef state.

Under a scenario whereby sea urchins rapidly adapt to conditions of OA, the present overgrazed reef-scape is poised to persist. In contrast, if sea urchins show nil adaptation and the refuge effect of adjacent kelp beds on local pH is insufficient, sea urchin abundance would collapse enabling kelp to dominate the reef-scape. Notably, the progression towards either of these two extremes would also likely manifest as patchiness in the reef-scape, albeit unstable patchiness, as the reef system transitions towards the alternative barrens or kelp dominated stable states [22]. Speculatively, even if more tolerant OA genotypes were to be selected for, the process of adaptation would likely involve temporary reductions in adult urchin population size (and therefore extent of barrens), as non-tolerant genes are thinned out [33]. Alternatively, in the absence of adaptation, the performance of the urchin population would gradually deteriorate as pH decreases thus enabling local kelp beds to re-establish.

Concurrent with projections of decline in the pH of Tasmanian coastal waters, warming by up to ~4°C from a mean of ~14°C to ~18°C by 2100 [32] is not projected to negatively affect either the kelp or the sea urchin species directly in this region. This is because both kelp *E*. *radiata* and the urchin *C*. *rodgersii* occur at the cool ‘leading-edge’ of their range in Tasmania, with optimal temperatures of 16–26°C [34] and 15–25°C [35] respectively. However, while warming appears poised to improve performance of kelp and urchins respectively, mesocosm studies have shown fast growing, opportunistic understory species or filamentous macroalgal turfs to become favoured under warmer lower pH waters; but which may require additional disturbances to intact kelp canopies to become dominant, such as storms, nutrient pollution, sedimentation, and/ or turbidity which are all expected to increase in future due to anthropogenic forcing [6, 36, 37]. This could potentially limit kelp recruitment and therefore patchiness in regrowth of macroalgae on barrens in future may involve novel macroalgal assemblages and altered rates of CO_2_ drawdown, as well as differences in the scales of local pH refugia for calcifiers on rocky reefs. Likewise, across the warm ‘trailing edges’ of kelp bed communities undergoing climate-driven retraction, e.g. northern New South Wales, tropicalization of temperate reef communities is increasing the abundance of herbivorous fish guilds that can further limit kelp recruitment and affect phase-shift from kelps to turfs to ultimately corals [38, 39], which will also alter patterns of CO_2_ drawdown across reefs in these regions [40].

Notably, the ability of kelp to act as a potential refuge against OA also appears context dependent as photosynthetic drawdown of CO_2_, and thus the refugia effect, would appear to be greatest in shallower kelp beds, particularly those in sheltered locations where there is greater seawater retention and capacity for kelp to alter seawater chemistry [12, 41]. This notion is further supported by the 0.40 units pH variability measured in other kelp beds in Tasmania around the same time as this study [20]. We also acknowledge that pH variability itself (which can either have no effect, or positive or negative effects on marine calcifiers [42]) was not captured in our projections of mean trends in pH across the kelp-barrens transition, due to a present lack of agreement on its effects. The majority of pH variability here was consistent with photosynthetic modification by kelp during the day, and their respiration at night [18, 19]. Furthermore, the patchiness of kelp habitats across future reef-scapes would further increase local variability in the pH environment which could have unknown consequences for the adaptive capacity of marine calcifiers [43]. Notably, unless urchins become locally depleted by severe impacts of OA (Scenario iii Fig 5B), then negative impacts of urchins on reef-based fisheries and biodiversity [44] are poised to continue as barrens will constitute approximately half of the reef scape even under the mosaicked future scenario (Fig 5Bii). Furthermore urchin grazing of the understory algae beneath intact kelp patches (offering refuge from OA), also negatively impacts species such as highly valued blacklip abalone *Haliotis rubra*, independent of complete kelp canopy loss [45].

## Conclusion

Our observations of differential patterns in pH across kelp beds and urchin barrens in combination with projections of future pH conditions, indicates a potentially important role for kelp as a pH refugia for sea urchins and other marine calcifying organisms under OA. Based on trajectories of pH by the end of this century, such kelp refugia are poised to not only alter local-scale herbivore-algae dynamics due to the potential for pH dependency of urchins on their kelp prey, but also lead to altered expression of phase-shift dynamics across reef-scapes. Under scenarios of nil-adaptation of functionally important marine calcifiers to low pH, monitoring changes and stability of habitat patchiness for reef systems therefore emerges as a possible indicator of ecosystem impacts of OA. Given the interactions between overfishing and ocean warming which has led to large increase in urchin abundance and cascading effects across reef-scapes in eastern Tasmania [8, 46], the possible ameliorating effect of kelps for myriad marine calcifiers including urchins themselves highlights that research on multiple anthropogenic impacts requires a holistic ecosystem-based approach. As such, effective climate change adaptation strategies will similarly necessitate ecosystem-based management. Ultimately, our research reveals that confronting OA in the world’s oceans requires understanding spatially complex feedbacks between primary producers, herbivores, and higher trophic groups which can be heavily impacted by pervasive non-climate stressors such as fishing.

## Materials and methods

### Measurements of pH

pH was measured within kelp beds and on urchin barren grounds using automated *in situ* loggers (7,362 logged pH measurements over a total of 82 days) and Niskin-bottle samples used to cross-calibrate *in situ* loggers (at start and end of each logger deployment) and to map the pH environment across the kelp-barrens interface at multiple sites (36 bottle samples in total). Automated *SeapHOx* sensors were programmed to read pH every 15 minutes and were deployed over 82 days yielding a total of 3,754 and 3,608 measurements in kelp bed and barren grounds respectively. Niskin-bottle mapping of pH along the kelp-barrens interface involved sampling along duplicate transects spanning the transition from kelp beds to urchin barren grounds at two sites: St. Helens Island (41°20'37.39"S; 148°20'32.13"E) and Sloop Rock (41°12'35.44"S; 148°17'37.79"E), see Fig 1. Kelp-barrens transects were established using vessel to achieve a straight transect perpendicular to shore which provided an optimal cross-section of the kelp bed to urchin barren ground transitions. Two replicate kelp-barrens transects were conducted at each site and were separated by approx. 300 metres. For each transect, the first bottle sample was taken over kelp beds close to the shoreline at 5 m depth. Subsequent samples were then taken perpendicularly to the shore every 10 m out to a distance of 30 m after which samples were taken every 20 m until the final sample was 140 m away from the first sample position. Niskin bottle seawater samples were transferred to 100 ml Perspex containers and poisoned with mercury chloride for measurement of pH later in the day. For these samples, pH was measured using a pH electrode (Thermo Scientific Orion 8107 BNUMD Ross Ultra pH/ATC Triode) attached to a pH meter (Thermo Scientific Orion Star A216 pH/RDO/DO meter) that was calibrated using TRIS and amp buffers prepared as per A. Dickson’s recommendations [47] at 14°C. All pH measurements will be referred to on the total scale throughout.

To obtain temporal variation in pH for kelp bed and urchin barrens, two SeapHOX loggers with Durafet solid state pH sensors were deployed from 29^th^ to 31^st^ October 2013, one at 5 m depth (kelp bed) and the other at 15 m (sea urchin barren). A second deployment within a deep kelp bed (15 m depth) and a sea urchin barren (15 m depth) occurred from 6^th^ June until 8^th^ August 2014. Both deployments were at St. Helens Island. The SeapHOXs recorded temperature, salinity, pH and oxygen concentration at half hourly intervals. An ENVCO pHTempion combined pH and temperature logger (calibrated as per above) was also deployed. For cross-calibration between the SeapHOXs and ENVCO, 250 ml bottle samples for total dissolved inorganic carbon (DIC) and total alkalinity (A_T_) were taken on the last and first days of deployment. All bottle samples were poisoned with mercuric chloride immediately following collection. DIC concentrations of bottle samples were determined using a SOMMA DIC analyser and 5011 UIC coulometer [47, 48]. An open cell potentiometric titration and a Metrohm 904 Titrando was used to measure total alkalinity [47]. Both DIC and A_T_ measurements were verified using analysis of Certified Reference Material from the Scripps Institution of Oceanography with an accuracy and precision of ± 2 micromol/kg for both parameters. The DIC and A_T_ data were used to calibrate the SeapHOx pH sensor [49].

### Sea urchin abundance and macroalgal composition

Three 15 m transects were visually assessed *in situ* by divers in both the shallow kelp band (5–6 m) and the sea urchin barrrens (12–17 m) on the 29^th^ October 2013 at St. Helens Island and the 30^th^ of October 2013 at Sloop Rock, and a deep kelp bed (15–16 m) was also surveyed on 8^th^ August 2014. Within each transect the number of *Ecklonia radiata* and *Phyllospora comosa* stipes were counted within 1 m of the 15 m transect line, and their percent cover in 5 × 1 m bands was recorded. These two species were the only large macroalgal species found in our surveys, while *E*. *radiata* was the only large macroalga defining the deep kelp beds. *Centrostephanus rodgersii* were also counted within the 1 m band. 10 photoquadrats (25 × 25 cm) were taken within each transect for determination of understorey macroalgal percent cover (for macroalgal community descriptions at both sites see S1 Table in S1 File).

### Ethics statement

No specific permissions were required for sampling water chemistry or visual assessment of these locations as sampling did not involve the collection of flora and/ or fauna.

### Analysis

Testing the null hypothesis of ‘no difference’ in pH between kelp beds and sea urchin barrens, a 2-way fixed effects analysis of variance (ANOVA; *n* = 3 replicate bottle samples randomly sub-sampling each habitat) was used to examine variability in pH across the factors of *Habitat* (kelp beds *vs*. urchin barrens) and *Depth* (shallow *vs*. deep, fixed factor) at St. Helens Island. In addition, a separate mixed effects 2-way ANOVA examined pH variability between *Site* (St. Helens Is. vs. Sloop Rock, random factor) and *Zone* (kelp beds *vs*. urchin barrens edge *vs*. urchin barren interior). Where transformation of the response variable was required to meet assumptions of homoscedasticity and normality, the appropriate stabilising transformation is shown and specified to the response variable *Y*. *RStudio* (Version 0.98.953 - © 2009–2013 *RStudio*, *Inc*.) was used for all statistical analyses (refer to S2 Table in S1 File).

### Future projections of pH and sea urchin thresholds across kelp bed/ barren ground interfaces

To calculate projected pH across the kelp bed to sea urchin barren transition zone under ocean acidification (using the business-as-usual CO_2_ emissions projection—RCP8.5), we used values measured across the transition zone in 2014 and factored for the increased capacity for kelp to alter pH as the ambient pH declines (due to increased CO_2_ uptake relative to HCO_3_^-^ [50]). Based on the RCP8.5 business-as-usual projection for this region [32], we examined dynamics in pH across the kelp-barrens zone for two different time horizons (year 2050 and 2100) relative to the pH thresholds defining reduced performance for sea urchins.

Sea urchin pH thresholds were determined from experimental data on fertilization and early development rates across pH for *Centrostephanus rodgersii*, plus data from other sea urchin species defining performance of juvenile and adult urchins across pH (S4 Fig in S1 File). Reduction in urchin performance was assumed to translate directly to reduced grazing on barren grounds to below the threshold required for maintenance of barrens (7). Furthermore, rapid local recovery of kelp beds (within 18 months in Tasmania (31)) occurs on barrens once grazing pressure falls below this critical threshold (7). Notably, *C*. *rodgersii* forms barrens as a coalescing network of grazed patches and local reductions in urchin density on barrens below the kelp recovery threshold allows kelp patches to re-establish given high localized fidelity of the urchin to crevice shelters (31). Projections of OA to 2100 indicate that the ‘acidifying’ pH environment on barren grounds will increasingly compromise the performance of grazing urchins on barrens grounds at distances beyond the localized remediating effect of kelp beds, which was estimated to be ~40 m based on projections of current day measurements. Conceptualizations of possible future reef-scapes under OA were developed, representing (1.) status quo whereby urchins adapt to low pH predicted for 2100, such that present day reefs remain unchanged, (2.) no adaptation by urchins but whereby kelp provides local refugia as modelled spatially by cellular automaton, (3.) no adaptation by urchins and insufficient kelp refuge from OA leading to a kelp dominated reef-scape. The 3 future models thus represent a nil-effect, moderate-effect (most likely based on measured pH relative to documented impacts on urchin performance), and an extreme-effect of OA on temperate reef ecosystems.

For the most likely moderate-effect model (based on observed measurements), a cellular automaton coded in Excel (simulating ‘Conway's Game of Life’ *after* https://danbscott.ghost.io/cellular-automata-in-excel/) was used to simulate the future reef configuration (based upon nil adaptation by urchins to OA) across a 640 m by 640 m reef-scape composed of 256 individual 40 m by 40 m cells. The present-day reef configuration of extensive barren grounds with kelp flanking the shallow margin (where wave action limits grazing urchins) was initially seeded by randomizing the transition of barren cells to kelp cells to simulate reduced performance of grazing urchins and thus local recovery of kelp patches on extensive barren grounds. Cellular patterns across the reef-scape were then allowed to emerge on their own following repeated application of the following 3 rules at each time-step, which approximated 18 months as required for kelp regrowth or overgrazing:

1. Any barren cell with fewer than 1 kelp cell neighbour (or >7 barren neighbours) is overgrown by kelp due to negative effect of OA on grazing urchins at distances >40 m from a kelp cell refuge (i.e. the “Overgrowth” rule);
2. Any barren cell with at least 1 kelp neighbour lives on to the next generation due to the local kelp refuge effect from OA at the scale of 40 m (i.e. the “Persistence” rule);
3. Any kelp cell with ≥7 barren neighbours becomes a barren cell due to urchin overgrazing (i.e. the “Overgrazing” rule).

Repeated application of the rules was achieved by repeatedly copying and pasting the cellular automaton grid within Excel, using relative cell referencing so that the previous reef-scape iteration was re-iterated (as opposed to iterating the initial seed reef-scape). Iterations of these 3 rules across randomly seeded reef-scapes revealed that stable reef-scape configurations were achieved within 10 iterations, at which point the configuration persisted indefinitely. Each iteration approximates 18 months based on the time for recovery of kelp beds in the absence of urchins [44], thus 10 iterations approximates simulation over 15 years. Conditional formatting was applied so that ‘living’ barren cells (“1”) were red and ‘dead’ barren cells, i.e. kelp cells (“0”), were green. The 8 possible configurations surrounding each cell (except boundary cells) and the state-dependent (barren vs kelp) rule, as applied to each central cell, can be seen in S5 Fig in S1 File.

## Supporting information

S1 File (DOCX)

S1 Data (XLSX)
