# Supplementary material for: Remnant kelp bed refugia and future phase-shifts under ocean acidification
Source: PLoS One. 2020 Oct 9;15(10):e0239136. doi: 10.1371/journal.pone.0239136 (PMC7546474; doi:10.1371/journal.pone.0239136)
Supplement: S1 File — (DOCX) [file pone.0239136.s001.docx]

Kelp bed refugia and future phase-shifts under acidifying seas

Scott D. Ling, Christopher E. Cornwall, Bronte Tilbrook, Catriona L. Hurd

Email: [Scott.Ling@utas.edu.au](mailto:Scott.Ling@utas.edu.au)

**This PDF file includes:**

S1 to S6 Figs

S1 to S2 Tables

References (specific to S4 Fig)

**S1 Fig.**

S2 Fig.

**S3 Fig.**

**S4 Fig.**

**S4 References**

List of references sourced to determine effects of Ocean Acidification on sea urchin life-history stages (S4 Fig).

| Asnaghi, V., Chiantore, M., Mangialajo, L., Gazeau, F., Francour, P., Alliouane, S. and Gattuso, J.P., 2013. Cascading effects of ocean acidification in a rocky subtidal community. *PloS one*, 8*,* 61978. |
| --- |
| Baggini, Cecilia, Issaris, Yannis, Salomidi, Maria, Hall-Spencer, Jason 2015. Herbivore diversity improves benthic community resilience to ocean acidification. *Journal of Experimental Marine Biology and Ecology*, 469, 98-104. |
| Brothers, C.J., Harianto, J., McClintock, J.B. and Byrne, M., 2016. Sea urchins in a high-CO2 world: the influence of acclimation on the immune response to ocean warming and acidification. *Proc. R. Soc. B,* 283, 20161501. |
| Byrne, M., Ho, M., Wong, E., Soars, N.A., Selvakumaraswamy, P., Shepard-Brennand, H., Dworjanyn, S.A. and Davis, A.R., 2011. Unshelled abalone and corrupted urchins: development of marine calcifiers in a changing ocean. *Proceedings of the Royal Society of London B: Biological Sciences*, 27*,* 2376-2383. |
| Byrne, M., Smith, A.M., West, S., Collard, M., Dubois, P., Graba-landry, A. and Dworjanyn, S.A., 2014. Warming influences Mg2+ content, while warming and acidification influence calcification and test strength of a sea urchin. Environmental science & technology, 48, 12620-12627. |
| Byrne, M., Soars, N., Selvakumaraswamy, P., Dworjanyn, S.A. and Davis, A.R., 2010. Sea urchin fertilization in a warm, acidified and high pCO 2 ocean across a range of sperm densities. *Marine Environmental Research*, 69*,* 234-239. |
| Calosi, P., Rastrick, S. P. S., Graziano, M., Thomas, S. C., Baggini, C., Carter, H. A., Hall-Spencer, J. M., Milazzo, M., Spicer, J. I. 2013. Distribution of sea urchins living near shallow water CO2 vents is dependent upon species acid-base and ion-regulatory abilities. *Marine Pollution Bulletin* 73, 470 - 484. |
| Catarino, A.I., Bauwens, M. and Dubois, P., 2012. Acid–base balance and metabolic response of the sea urchin Paracentrotus lividus to different seawater pH and temperatures. *Environmental Science and Pollution Research*, 19*,* 2344-2353. |
| Collard, M., Rastrick, S.P., Calosi, P., Demolder, Y., Dille, J., Findlay, H.S., Hall-Spencer, J.M., Milazzo, M., Moulin, L., Widdicombe, S. and Dehairs, F., 2015. The impact of ocean acidification and warming on the skeletal mechanical properties of the sea urchin *Paracentrotus lividus* from laboratory and field observations. *ICES Journal of Marine Science*, 73,727-738. |
| Dery, A., Collard, M. and Dubois, P., 2017. Ocean acidification reduces spine mechanical strength in Euechinoid but not in Cidaroid sea urchins. *Environmental Science & Technology*, 51, 3640-3648. |
| Doo, S.S., Dworjanyn, S.A., Foo, S.A., Soars, N.A. and Byrne, M., 2011. Impacts of ocean acidification on development of the meroplanktonic larval stage of the sea urchin Centrostephanus rodgersii. *ICES Journal of Marine Science*, 69*,* 460-464. |
| Dupont, S., Dorey, N., Stumpp, M., Melzner, F., Thorndyke, M. 2013. Long-term and trans-life-cycle effects of exposure to ocean acidification in the green sea urchin *Strongylocentrotus droebachiensis*. *Marine Biology*, 160, 1835-1843. |
| Emerson, C.E., Reinardy, H.C., Bates, N.R. and Bodnar, A.G., 2017. Ocean acidification impacts spine integrity but not regenerative capacity of spines and tube feet in adult sea urchins. *Royal Society Open Science*, 4, 170140. |
| Figueiredo, D.A.L., Branco, P.C., dos Santos, D.A., Emerenciano, A.K., Iunes, R.S., Borges, J.C.S. and da Silva, J.R.M.C., 2016. Ocean acidification affects parameters of immune response and extracellular pH in tropical sea urchins *Lytechinus variegatus* and *Echinometra luccunter*. *Aquatic Toxicology*, 180, 84-94. |
| Foo, S.A., Dworjanyn, S.A., Poore, A.G. and Byrne, M., 2012. Adaptive capacity of the habitat modifying sea urchin *Centrostephanus rodgersii* to ocean warming and ocean acidification: performance of early embryos. *PLoS One*, 7*,* 42497. |
| García, E., Clemente, S. and Hernández, J.C., 2015. Ocean warming ameliorates the negative effects of ocean acidification on *Paracentrotus lividus* larval development and settlement. *Marine Environmental Research*, 110, 61-68. |
| García, E., Hernández, J.C., Clemente, S., Cohen-Rengifo, M., Hernández, C.A. and Dupont, S., 2015. Robustness of *Paracentrotus lividus* larval and post-larval development to pH levels projected for the turn of the century. *Marine Biology*, 162*,* 2047-2055. |
| Hazan, Y., Wangensteen, O.S., and Fine, M. 2014. Tough as a rock-boring urchin: adult Echinometra sp EE from the Red Sea show high resistance to ocean acidification over long-term exposures. *Marine Biology*, 161, 2531-2545. |
| Kroeker, K.J., Gambi, M.C. and Micheli, F., 2013. Community dynamics and ecosystem simplification in a high-CO2 ocean. *Proceedings of the National Academy of Sciences*, 110, 12721-12726. |
| Kurihara, H., Yin, R., Nishihara, G.N., Soyano, K. and Ishimatsu, A., 2013. Effect of ocean acidification on growth, gonad development and physiology of the sea urchin *Hemicentrotus pulcherrimus*. *Aquatic Biology*, 18, 281-292. |
| Manríquez, P.H., Torres, R., Matson, P.G., Lee, M.R., Jara, M.E., Seguel, M.E., Sepúlveda, F. and Pereira, L., 2017. Effects of ocean warming and acidification on the early benthic ontogeny of an ecologically and economically important echinoderm. *Marine Ecology Progress Series*, 563*,* 169-184. |
| Miles, H., Widdicombe, S., Spicer, J.I. and Hall-Spencer, J., 2007. Effects of anthropogenic seawater acidification on acid–base balance in the sea urchin *Psammechinus miliaris*. *Marine Pollution Bulletin*, 54, 89-96. |
| Moulin, L., Grosjean, P., Leblud, J., Batigny, A. and Dubois, P., 2014. Impact of elevated pCO2 on acid–base regulation of the sea urchin *Echinometra mathaei* and its relation to resistance to ocean acidification: a study in mesocosms. *Journal of experimental marine biology and ecology*, 457, 97-104. |
| Moulin, L., Grosjean, P., Leblud, J., Batigny, A., Collard, M. and Dubois, P., 2015. Long-term mesocosms study of the effects of ocean acidification on growth and physiology of the sea urchin *Echinometra mathaei*. *Marine environmental research*, 103, 103-114. |
| Nasuchon, N., Hirasaka, K., Yamaguchi, K., Okada, J. and Ishimatsu, A., 2017. Effects of elevated carbon dioxide on contraction force and proteome composition of sea urchin tube feet. *Comparative Biochemistry and Physiology Part D: Genomics and Proteomics*, 21, pp.10-16. |
| Pecorino, D., Barker, M.F., Dworjanyn, S.A., Byrne, M. and Lamare, M.D., 2014. Impacts of near future sea surface pH and temperature conditions on fertilisation and embryonic development in *Centrostephanus rodgersii* from northern New Zealand and northern New South Wales, Australia. *Marine Biology*, 161*,* 101-110. |
| Rodríguez, A., Hernández, J.C., Brito, A. and Clemente, S., 2017. Effects of ocean acidification on juveniles sea urchins: Predator-prey interactions. *Journal of Experimental Marine Biology and Ecology*, 493, 31-40. |
| Shirayama, Y. and Thornton, H., 2005. Effect of increased atmospheric CO2 on shallow water marine benthos. *Journal of Geophysical Research: Oceans*, 110(C9). |
| Spicer, J.I., Widdicombe, S., Needham, H.R. and Berge, J.A., 2011. Impact of CO 2-acidified seawater on the extracellular acid–base balance of the northern sea urchin *Strongylocentrotus droebachiensis*. *Journal of Experimental Marine Biology and Ecology*, 407, 19-25. |
| Stumpp, M., Trübenbach, K., Brennecke, D., Hu, M.Y. and Melzner, F., 2012. Resource allocation and extracellular acid–base status in the sea urchin *Strongylocentrotus droebachiensis* in response to CO 2 induced seawater acidification. *Aquatic Toxicology*, 110, 194-207. |
| Suckling, C.C., Clark, M.S., Beveridge, C., Brunner, L., Hughes, A.D., Harper, E.M., Cook, E.J., Davies, A.J. and Peck, L.S., 2014. Experimental influence of pH on the early life-stages of sea urchins II: increasing parental exposure times gives rise to different responses. *Invertebrate Reproduction & Development*, 58, 161-175. |
| Uthicke, S., Soars, N., Foo, S. and Byrne, M., 2013. Effects of elevated pCO2 and the effect of parent acclimation on development in the tropical Pacific sea urchin *Echinometra mathaei*. *Marine Biology*, 160*,* 1913-1926. |
| Wangensteen, O.S., Dupont, S., Casties, I., Turon, X. and Palacín, C., 2013. Some like it hot: temperature and pH modulate larval development and settlement of the sea urchin *Arbacia lixula*. *Journal of experimental marine biology and ecology*, 449, 304-311. |
| Wolfe, K., Dworjanyn, S.A. and Byrne, M., 2013. Effects of ocean warming and acidification on survival, growth and skeletal development in the early benthic juvenile sea urchin (*Heliocidaris erythrogramma*). *Global Change Biology*, 19, 2698-2707. |

**S5 Fig.**


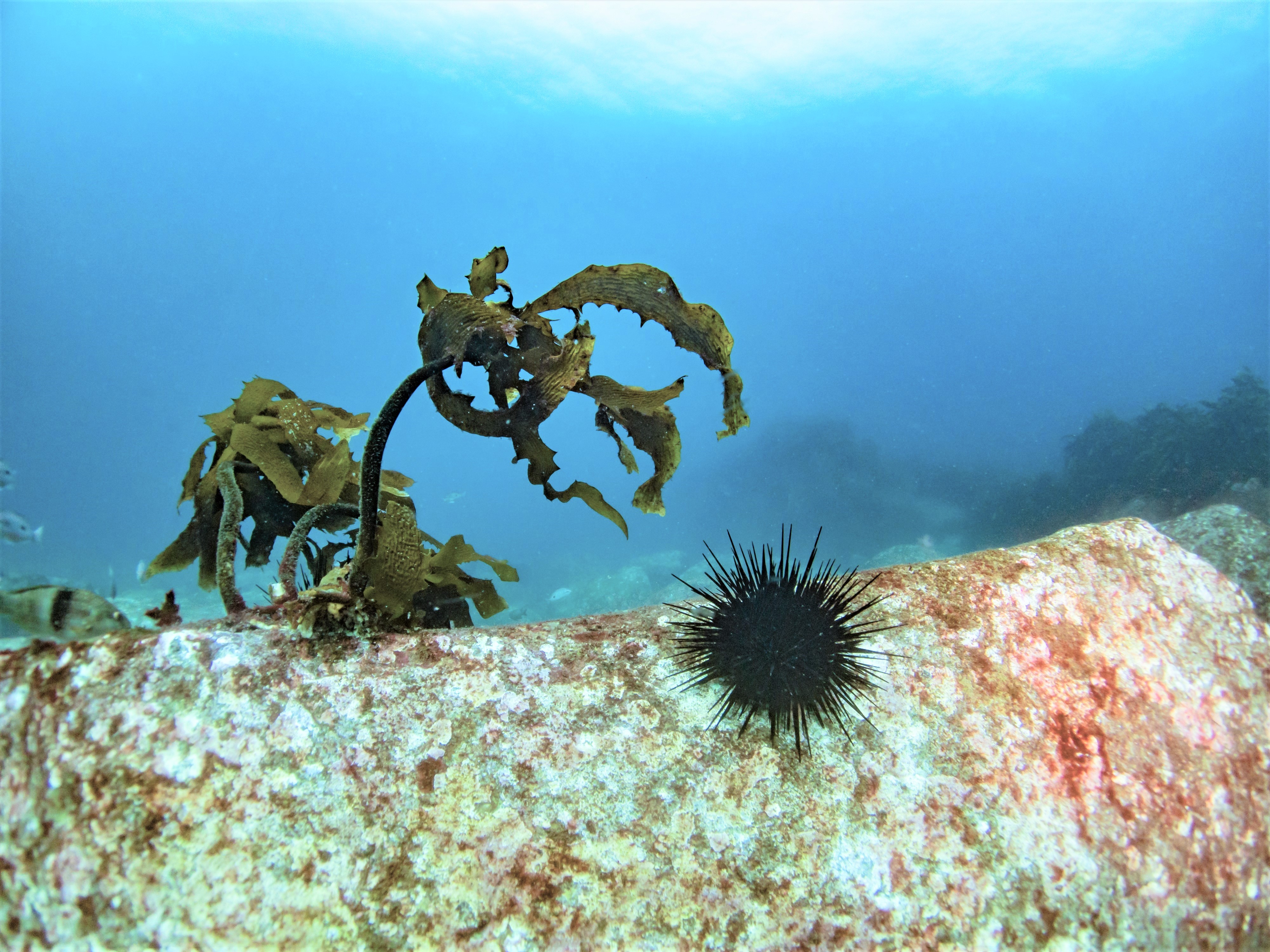


**S6 Fig.**

**S1 Table.**

S2 Table.

| A | | | | | | | | | | |  | | | | | | |  |  |  |  |  |  |  |
| --- | --- | --- | --- | --- | --- | --- | --- | --- | --- | --- | --- | --- | --- | --- | --- | --- | --- | --- | --- | --- | --- | --- | --- | --- |
| Source | Df | Sum Sq | | | Mean Sq | | | | F value | | | | | | Pr(>F) | | | |  |  |  |  |  |  |
| Habitat (fixed) | 1 | 0.000938 | | | 0.000938 | | 7.166 | | | | | | **0.0145** | | | | | |  |  |  |  |  |  |
| Depth (fixed) | 1 | 4.2E-06 | | | 4.2E-06 | | 0.032 | | | | | | 0.8602 | | | | | |  |  |  |  |  |  |
| Habitat*Depth (fixed) | 1 | 3.75E-05 | | | 3.75E-05 | | 0.287 | | | | | | 0.5983 | | | | | |  |  |  |  |  |  |
| Residuals | 20 | 0.002617 | | | 0.000131 | |  | | | | | |  | | | | | |  |  |  |  |  |  |
| \|  \| \| --- \|   B | | | |  | | | | | | | |  | | | | | | | |  |  |  |  |  |
| Source | Df | | Sum Sq | | | Mean Sq | | | | F value | | | | | | Pr(>F) | | | | |  | |  |  |
| Habitat (fixed) | 2,2 | | 0.030872 | | | 0.015436 | | 99.232 | | | | | | **0.0234** | | |  | | | | |  |  |  |
| Site (random) | 1 | | 0.002844 | | | 0.002844 | | 18.286 | | | | | | **0.0002** | | |  | | | | |  |  |  |
| Habitat*Site (random) | 2 | | 0.000739 | | | 0.000369 | | 2.375 | | | | | | 0.1103 | | |  | | | | |  |  |  |
| Residuals | 30 | | 0.004667 | | | 0.000156 | |  | | | | | |  | | | | | | |  | | |  |
|  | | | | | | | | | | | | | | | | | | | | | | | | |
